# MASTL inhibition promotes mitotic catastrophe through PP2A activation to inhibit cancer growth and radioresistance in breast cancer cells

**DOI:** 10.1186/s12885-018-4600-6

**Published:** 2018-07-05

**Authors:** Yi Na Yoon, Min Ho Choe, Kwan-Young Jung, Sang-Gu Hwang, Jeong Su Oh, Jae-Sung Kim

**Affiliations:** 1 Division of Radiation Cancer Research, Korea Institute of Radiological and Medical Sciences, 215-4 Gongneung-Dong, Nowon-Ku, Seoul, 139-706 South Korea; 2 Radiological and Medico-Oncological Sciences, University of Science and Technology, Daejeon, South Korea; 3 Department of Life Sciences and Biotechnology, College of Life Science and Biotechnology, Korea University, Seoul, South Korea; 4 Center for Medicinal Chemistry, Korea Research Institute of Chemical Technology, Daejeon, South Korea; 5 Department of Integrative Biotechnology, Sungkyunkwan University, Suwon, South Korea

**Keywords:** MASTL, Mitotic catastrophe, Radiation, Breast cancer

## Abstract

**Background:**

Although MASTL (microtubule-associated serine/threonine kinase-like) is a key mitotic kinase that regulates mitotic progression through the inactivation of tumor suppressor protein phosphatase 2A (PP2A), the antitumor mechanism of MASTL targeting in cancer cells is still unclear.

**Methods:**

MASTL expression was evaluated by using breast cancer tissue microarrays and public cancer databases. The effects of MASTL depletion with siRNAs were evaluated in various breast cancer cells or normal cells. Various methods, including cell viability, cell cycle, soft agar, immunoblotting, immunofluorescence, PP2A activity, live image, and sphere forming assay, were used in this study.

**Results:**

This study showed the oncosuppressive mechanism of MASTL targeting that promotes mitotic catastrophe through PP2A activation selectively in breast cancer cells. MASTL expression was closely associated with tumor progression and poor prognosis in breast cancer. The depletion of MASTL reduced the oncogenic properties of breast cancer cells with high MASTL expression, but did not affect the viability of non-transformed normal cells with low MASTL expression. With regard to the underlying mechanism, we found that MASTL inhibition caused mitotic catastrophe through PP2A activation in breast cancer cells. Furthermore, MASTL depletion enhanced the radiosensitivity of breast cancer cells with increased PP2A activity. Notably, MASTL depletion dramatically reduced the formation of radioresistant breast cancer stem cells in response to irradiation.

**Conclusion:**

Our data suggested that MASTL inhibition promoted mitotic catastrophe through PP2A activation, which led to the inhibition of cancer cell growth and a reversal of radioresistance in breast cancer cells.

**Electronic supplementary material:**

The online version of this article (10.1186/s12885-018-4600-6) contains supplementary material, which is available to authorized users.

## Background

Mitosis is the most dynamic phase of the cell cycle, in which one cell gives rise to two genetically identical daughter cells, and involves diverse cellular changes, such as dynamic reorganization of cellular structures, including chromosomes, nucleus, ER, and Golgi apparatus [1, 2]. It is therefore regarded as the most fragile phase of the cell cycle, during which cancer cells exposed to various insults are highly susceptible to cell death [2]. Many cancer therapies, such as radiation and paclitaxel, mostly perturb mitotic progression with prolonged mitotic arrest, which leads to tumor regression [1, 2]. Compared to cancer cells, normal cells are generally more tolerant to antimitotic drugs owing to the existence of various surveillance mechanisms and presence of fewer mitotic cells. For example, the inhibition of PLK1, a major mitotic kinase, selectively eradicates cancer cells, but not normal cells, through mitotic cell death [3, 4]. Therefore, the targeting of mitosis has been considered an attractive therapeutic strategy for selective anticancer treatment.

Mitotic catastrophe is a type of cell death that results from abnormal mitosis following DNA damage, such as that caused by irradiation, and is related to premature chromosome condensation and the formation of large cells with multiple micronuclei [5, 6]. DNA damage-induced mitotic catastrophe can be driven by the activation of caspases, particularly caspase-2 [6, 7]. Recently, it was shown that the activation of caspase-2 in response to DNA damage plays an oncosuppressive role through the reduction of genomic instability and chromosomal aberrations that may promote tumorigenesis [6]. In addition, mitotic catastrophe is known to be the primary form of cell death induced by radiotherapy in cancer [5] and that the evasion of mitotic catastrophe is associated with tumor resistance to anticancer treatments in several cancer cells [8]. Furthermore, it is well established that cancer stem cells play a key role in tumor radioresistance through the modulation responses to DNA damage [9]. Nevertheless, the regulatory factors of mitotic catastrophe and cancer stem cells in radioresistance are largely unknown.

MASTL (microtubule-associated serine/threonine kinase-like), also known as Greatwall kinase, was first identified in Drosophila as a key mitotic kinase involved in chromosome condensation and mitotic progression [1, 10]. MASTL acts as a regulator of mitotic progression through the phosphorylation of α-endosulfine (ENSA) and/or cAMP-regulated phosphoprotein 19, which in turn inactivates the protein phosphatase 2A complex (PP2A-B55) [11, 12]. The inactivation of PP2A-B55 is essential for the maintenance of cyclin B1/Cdk1 activity during normal mitosis. Mitosis is frequently upregulated in cancer cells and several studies have reported the upregulation of MASTL in various cancer tissues, including breast, head and neck, and colorectal cancers; hence, MASTL inhibition reduces tumor growth in vitro and in vivo [13–16]. Furthermore, recent reports suggested that MASTL regulates mitotic re-entry in response to DNA damage [13, 17, 18] and promotes cell transformation through the activation of Akt in human malignancies [14], which suggest that MASTL targeting may be an attractive strategy for anticancer treatment. However, the oncosuppressive mechanisms of MASTL targeting in tumor treatment and resistance are still unclear.

In this study, we observed that MASTL was upregulated and associated with poor prognosis in breast cancer tissues. MASTL depletion reduced the oncogenic properties through the selective induction of mitotic catastrophe in breast cancer cells and not in non-transformed normal cells. Furthermore, MASTL depletion increased the radiosensitivity of breast cancer cells and reduced the formation of the radioresistant breast cancer stem cells. Interestingly, we found that MASTL inhibition activated PP2A during these processes. Hence, our data provide evidence for an oncosuppressive mechanism through MASTL targeting that promotes mitotic catastrophe through PP2A activation in the treatment of breast cancer cells, including by radiotherapy.

## Methods

### Cell culture and treatment

All cell lines were purchased from American Type Culture Collection (ATCC; Manassas, VA). As indicated in the provided information, all cell lines were authenticated by their karyotypes, images, and detailed gene expression. The cell lines were preserved and passaged for less than 2 months in accordance with ATCC protocols and tested for mycoplasma infection by a PCR method once per week. MCF7, T47D, BT474, SKBR3, and HDFn cells were cultured in DMEM (Corning, NY) and HCC1937, HCC38, and MDA-MB-231 cells were cultured in RPMI (Corning). HUVECs were cultured in Medium 199 (Sigma, MO) containing 5 U/ml heparin (Sigma) and 10 ng/ml human recombinant basic fibroblast growth factor (Peprotech, NJ). All media types were supplemented with 10% fetal bovine serum (Corning) and 1% penicillin/streptomycin, and maintained in a humidified 5% CO_2_ incubator at 37 °C. The radioresistant CD44^high^/CD24^low^ MCF7 cells were established by using a previously described method [19, 20]. Briefly, CD44^+^/CD24^−^ subpopulations from MCF7 cells were isolated by their surface markers using a FACS Aria II (BD Biosciences, San Diego, CA). The radioresistant phenotype was determined by colony and sphere forming assays in response to irradiation. The cells were irradiated by using a ^137^cesium (Cs) ray source (Atomic Energy of Canada Ltd., Mississauga, Canada) at a dose rate of 3.81 Gy/min. A pan-caspase inhibitor, zVAD (20 μM; R&D systems, MN), was used to inhibit caspases. Prior to the experiments, cell number and viability were measured using a Luna II automated cell counter (Logos Biosystems, Anyang, Korea).

### Tissue samples

Forty-six pairs of breast cancer tissues and their matched non-tumor adjacent tissues were acquired from patients undergoing surgery for breast cancer at Korea University Hospital (Seoul, Korea) and Busan National University Hospital (Busan, Korea). All tissues were immediately frozen and stored in liquid nitrogen until RNA extraction. All patients gave signed, informed consent for their participation in scientific research (IRB number: K-1504-002-044).

### RNA isolation and qRT-PCR

RT-PCR was performed in accordance with a previously described protocol [20, 21]. Briefly, total RNA was isolated by using Qiazol reagents (Qiagen, Hilden, Germany) was reverse-transcribed by using the ImProm-II™ reverse transcription system (Promega, WI). Quantitative RT-PCR was performed on a Chromo 4 Cycler (Bio-Rad, CA) by using SYBR Premix Ex Taq (Takara Bio, Kyoto, Japan). The following PCR primers were used: MASTL (annealing temperature: 60 °C), forward 5′-CAGCATAGTGAAGCCCATTAGC-3′ and reverse 5′-GGATGTTTGCAGGGTCTTG-3′; PLK1, forward 5′-GATCTCGAGCTATGAGTGCTGCAGTGACTGCAG-3′ and reverse 5′-CGCGGTACCTTAGGAGGCCTTGAGACGGTTGC-3′; glyceraldehyde 3-phosphate dehydrogenase (GAPDH), forward 5′-CATCTCTGCCCCCTCTGCTGA-3′ and reverse 5′-GGATGACCTTGCCCACAGCCT-3′.

### Survival analysis

The survival analysis of MASTL in breast cancer was performed by using the PROGgene V2 Prognostic Database (http://watson.compbio.iupui.edu/chirayu/proggene/database/?url=proggene) [22]. Each analysis used “breast cancer” as the cancer type, “death,” “relapse,” or “metastasis” as survival measure, and bifurcated the gene expression at the median.

### Plasmids

Mouse MASTL cDNA was amplified by using PCR and cloned into the pcDNA3.1 vector. This construct was transfected by using TransIT 2020 (Mirus Bio, Madison, WI) to induce the ectopic overexpression of mouse MASTL. MCF7 cells (7.5 × 10^5^) were seeded in a 60-mm dish with siRNA assembled by G-fectin. After the cells were left to adhere to the dish for 6 h, 2 μg of mMASTL-pcDNA3.1 or empty-pcDNA3.1 vector applied to TransIT 2020 was added to the cells.

### RNA interference

The following constructs were used for RNA interference: MASTL.1, 5′-AAUGCCUGUGAAGUGUCUAACUU-3′; MASTL.2, 5′-CUAAUGAGGGUCAUAUUAAUU-3′; MASTL.3, 5′-CUUUGAAUAGAGAUAUUAAUU-3′; MASTL.4, 5′-GCCUUAUUCUAGCAAAUUAUU-3′; and MASTL.5, 5′-GAAUGAACUUGCAUAAUUAUU-3′; PP2A-Aα, 5′-GCAUCAAUGUGCUGUCAUATT-3′; and PP2A-Aβ, 5′-CGACUCAACAGUAUUAAGATT-3′. Non-silencing siRNA (Bioneer, Daejeon, Korea) was used as a negative control. The transfection of siRNAs (20 nmol/l Ctrl, 20 nmol/l MASTL, and 5 nmol/l PP2A siRNA) was performed by using G-fectin (Genolution, Seoul, Korea) in accordance with the manufacturer’s protocol.

### Cell viability assays

Cell viability was determined by the various assays, including the trypan blue assay, WST-8 assay, and FACS analysis, dependent on the experiments. Briefly, trypan blue exclusion assay was used to assess the effects of siRNA transfection. The numbers of viable cells were counted using LUNA-II™ (Automated cell counter; Logos Biosystem, VA). The WST-8 assay was used to investigate various inhibitors. Cell proliferation or viability was determined by using the WST-8 assay (Cyto X™ cell viability assay kit; LPS solution, Daejeon, Korea) in accordance with the manufacturer’s protocol. The absorbance was measured at 450 nm by using a Versamax microplate reader (Molecular Devices, CA). FACS analysis was used to examine the proliferation of transfected and irradiated cells by using a BD Accuri™ C6 (BD Biosciences).

### Cell cycle analysis

Cell cycle analysis was performed in accordance with a previously described protocol [20]. Briefly, the cells were treated with the indicated conditions, trypsinized, washed twice in PBS, and fixed with ice-cold 70% ethanol. Fixed cells were incubated with 50 μg/ml propidium iodide and 100 μg/ml RNase at 37 °C for 30 min and then analyzed by using a FACScalibur flow cytometer (BD Biosciences).

### Soft agar assay

The cells were transfected with 20 nmol/l control siRNA or 20 nmol/l MASTL.5 siRNA. After 48 h, the cells were trypsinized, gently mixed with 0.45% agar medium mixture (Difco Noble Agar; BD Biosciences, CA), and re-seeded on 6-well plates covered with a layer of 0.9% agar in DMEM. After 2 weeks, the colonies were stained with 0.2% crystal violet (Sigma) and quantified by colony counting.

### Sphere formation assay

The cells were transfected with 20 nmol/l control siRNA or 20 nmol/l MASTL.5 siRNA. After 48 h, 20,000 cells were plated on ultra-low attachment plates (Corning) in serum-free DMEM-F12 medium (Gibco-BRL, MA) supplemented with B-27 (1:50; Invitrogen, CA), 20 ng/mL FGF (R&D Systems), and 20 ng/mL epidermal growth factor (R&D Systems). After 10–14 days, the sphere colonies were fixed with methanol. The inhibition rate of mammosphere formation of the control and MASTL-depleted cells was assessed by the average number of sphere colonies and their diameter (> 110 sphere colonies per data point). The average number of sphere colonies and their diameter were analyzed by using ImageJ software. Sphere colonies with a diameter below 50 mm were excluded from the analysis.

### Western blot analysis

Western blotting was performed in accordance with a previously described method [20, 21]. Briefly, the proteins were separated by using SDS-polyacrylamide gel electrophoresis, transferred to a nitrocellulose membrane, and detected through incubation with specific antibodies. The following antibodies were used: rabbit polyclonal anti-MASTL (Abgent, San Diego, CA), rabbit phospho-ENSA (Ser67)/ARPP19(Ser62) rabbit polyclonal ENSA, rabbit monoclonal anti-PLK1, rabbit monoclonal anti-cleaved PARP (Cell Signaling Technology, MA), rabbit polyclonal anti-phospho-H3 (Merck, NJ), rabbit monoclonal anti-γ-H2AX (Millipore, MA), and mouse polyclonal anti-β-actin (Santa Cruz Biotechnology, CA), and mouse monoclonal anti-caspase-2 (Cell Signaling Technology). The blots were developed by using peroxide-conjugated secondary antibody and an enhanced chemiluminescence detection system (Amersham Life Science, IL) and images of the bands were obtained by using an Amersham Imager 600 system (GE Healthcare).

### Immunofluorescence

Immunofluorescence analysis was performed in accordance with a previously described protocol [21, 23]. Briefly, the cells were fixed with 4% paraformaldehyde, permeabilized, and blocked with 0.1 Triton X-100 and 5% fetal calf serum in PBS. The fixed cells were consecutively incubated with primary antibodies against phospho-H3 (Millipore; 1:500), γ-H2AX (Millipore; 1:200), acetyl-tubulin (Sigma; 1:300), cleaved PARP (Cell Signaling Technology; 1:100), and secondary antibodies, such as anti-mouse Alexa-488 and anti-rabbit Alexa-594 (Molecular Probes, Eugene, OR; 1:200). The slides were mounted in DAPI-containing medium and images of the bands were then obtained by using a confocal laser-scanning microscope (LSM 710; Carl Zeiss, Inc.).

### Immunohistochemistry

Immunofluorescence analysis was performed in accordance with a previously described protocol [23]. Briefly, commercially available human tissue microarrays were purchased (SuperBioChips, Seoul, Korea). Tissue arrays were able to analyze up to 79 breast cancer specimens in addition to several matched normal tissues. Immunohistochemical staining was performed by using anti-MASTL rabbit polyclonal antibody (Abgent; 1:100). Immunostaining was detected by the avidin-biotin-peroxidase method in accordance with the manufacturer’s instructions (Invitrogen). The staining intensity was scored on a scale of 0–3: 0, no visible staining; 1+, faint staining; 2+, moderate staining; and 3+ (strong staining; Additional file 1: Figure S1A).

### PP2A activity assay

PP2A activity was assayed by using a previously described method [21, 23]. Briefly, the cell lysates were precipitated with an anti-PP2A-Aα/β antibody (Santa Cruz) and the immunocomplexes were collected by using protein A sepharose beads. One-quarter of the washed beads were used for western blotting to confirm PP2A immunoprecipitation efficiency. The remaining beads were used for the analysis of PP2A activity by using a RediPlate 96 EnzChek Serine/Threonine Phosphatase assay kit (Invitrogen) in accordance with the manufacturer’s protocol. Okadaic acid (OA; 50 nmol/l; Sigma) was used as a positive control for the inhibition of PP2A activity.

### Live imaging

The cells were transfected with 20 nmol/l control siRNA or 20 nmol/l MASTL.5 siRNA. After 24 h, time-lapse images were acquired at 6-min intervals by using confocal and phase-contrast microscopy (LSM 710; Carl Zeiss, Inc.).

### Statistical analysis

The two-tailed Student’s *t*-test was performed to analyze statistical differences between groups. *P*-values of less than 0.05 were considered to be statistically significant. Statistical analyses were computed by using Excel and XLSTAT software.

## Results

### MASTL is associated with tumor progression and poor prognosis in breast cancer

**Fig. 1:**
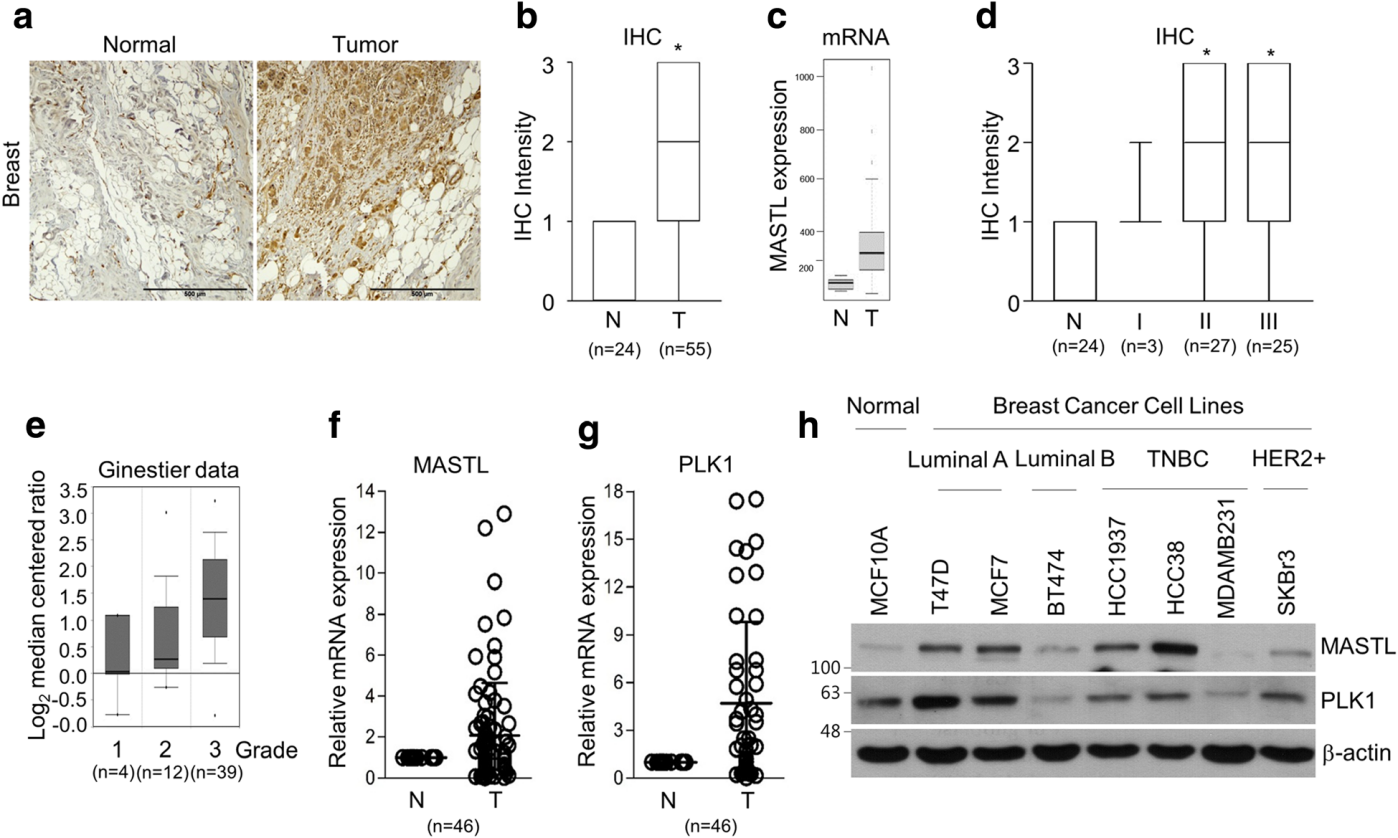
MASTL is associated with tumor progression in breast cancer. **a** Representative microscopic images of breast cancer and normal tissue counterparts stained with anti-MASTL antibody (left panels). Scale bars = 500 μm. **b**, **d** Quantified staining intensity of MASTL in breast cancers (*n* = 55) and normal tissues (*n* = 24). Staining intensity was scored as follows: 0, no staining; + 1, weak; + 2, moderate; and + 3, strong. *P < 0.05 compared with staining intensity of normal tissues. **c** MASTL mRNA expression in normal and breast cancer tissues was analyzed by using the GENT database (medicalgenome.kribb.re.kr/GENT). **e** MASTL mRNA expression in different breast cancer grades was analyzed by using the Oncomine database (http://www.oncomine.org) with the Ginestier dataset. The data are presented as box-and-whisker plots **b**, **c**, **d**, **e**. **f** MASTL and **g** PLK1 mRNA expression in breast cancer tissues and their normal counterparts (*n* = 46). **h** The cells were analyzed by immunoblotting for anti-MASTL and anti-PLK1 antibodies. The data represent typical results and are presented as the mean ± standard deviation of three independent experiments

To confirm the clinical relevance of MASTL targeting in breast cancer, we first evaluated MASTL expression by using breast cancer tissue microarrays and public cancer databases. As reported elsewhere [13, 14, 16], the protein and mRNA levels of MASTL were highly overexpressed in breast cancer tissues compared to in normal tissue counterparts (Fig. 1a-c) and MASTL was associated with breast cancer progression (Fig. 1d, e), as determined by the analysis of 79 breast cancer specimens (Fig. 1a, b, and d) and a public database (Fig. 1c and e). Furthermore, we confirmed that the mRNA levels of MASTL and PLK1 were overexpressed in breast cancer tissues compared to in their normal counterparts, as determined by the analysis of 46 pairs of breast cancer tissues and their matched non-tumor adjacent tissues, (Fig. 1f, g) and in various subtypes of breast cancer cell lines compared with normal breast cells (Fig. 1h), respectively, which indicated that MASTL expression may be associated with the mitotic activity of cancer cells. Further, we confirmed that high MASTL expression was correlated with poor levels of overall, recurrence-free, and metastasis-free survival in comparison with breast cancers with low MASTL expression (Additional file 1: Figure S1). Thus, these results indicated the clinical significance of MASTL targeting in breast cancer.

### MASTL depletion induces cell death in breast cancer cells

**Fig. 2:**
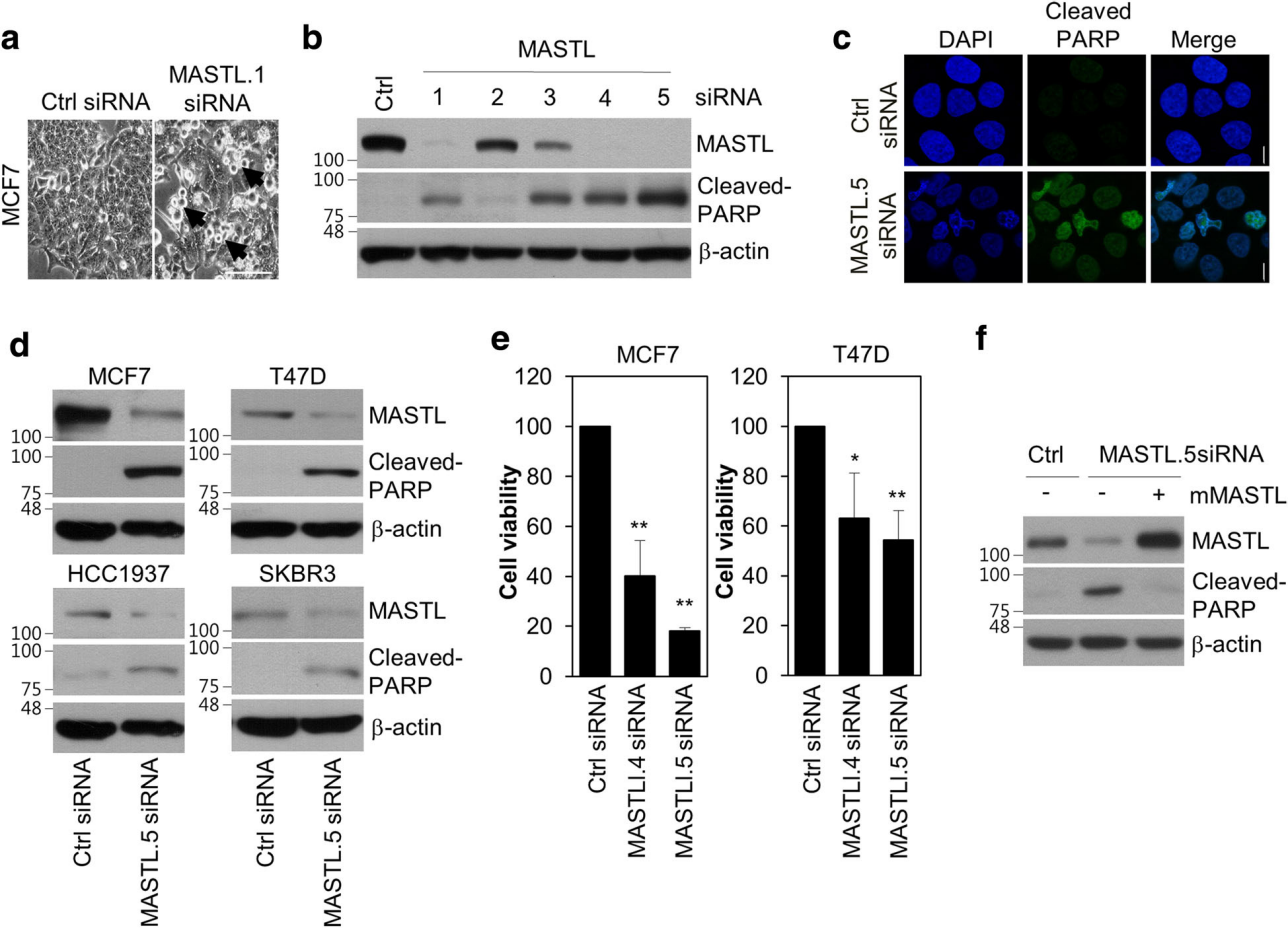
MASTL depletion induces cell death in breast cancer cells. The cells were transfected with either the control (Ctrl) siRNA or MASTL siRNAs, as indicated, for 48 h. **a** Representative light microscopy images of control (Ctrl) or MASTL.1 siRNA-depleted cells. Scale bar = 100 μm. **b** The cells were analyzed by immunoblotting or **c** immunofluorescence analysis with the indicated antibodies. Scale bars = 10 μm. **d** MCF7, T47D, HCC1937, and SKBR3 cells treated with either control (Ctrl) siRNA or MASTL.5 siRNA were analyzed by immunoblotting with the indicated antibodies. **e** The trypan blue exclusion assay was used to assess the effect of the cells treated with either control (Ctrl) siRNA, MASTL.4 siRNA, or MASTL.5 siRNA. **f** MCF7 cells were transfected with either control (Ctrl) siRNA, MASTL.5 siRNA, or both MASTL.5 siRNA and mouse MASTL vector for 48 h and then analyzed by immunoblotting for the indicated antibodies. The data represent typical results and are presented as the mean ± standard deviation of three independent experiments; **P < 0.01 and *P < 0.05

As MASTL inhibition reduces cell viability in various cancer cells [13–15] and targeting mitotic kinases such as PLK1 preferentially induces apoptosis in cancer cells compared to in non-transformed normal cells [3, 4], we expected that the inhibition of MASTL would induce cell death selectively in breast cancer cells and not in non-transformed normal cells. To examine this, we depleted MASTL in MCF7 cells by using siRNA #1, as described in a previous report [12], and observed the apoptotic phenotype of the MASTL-depleted cells (Fig. 2a). Subsequently, to make selective siRNAs to target MASTL, we designed four additional siRNAs that targeted the different regions of MASTL mRNA. SiRNA #1, #4, and #5 were able to efficiently knockdown MASTL expression and induce cell death of MCF7 cells (Fig. 2b). Among them, siRNA #5, which targeted the 5′ UTR region of MASTL mRNA, caused the most significant induction of cell death (Fig. 2b); this apoptotic phenotype was confirmed by immunofluorescence analysis, which showed an increase in cleaved-PARP (Fig. 2c). Next, we checked whether MASTL depletion induced cell death in several breast cancer cell lines, including MCF7, T47D, HCC1937, and SKBR3 cells. We found that MASTL depletion predominantly induced cell death and reduced cell viability in breast cancer cell lines, such as MCF7 and T47D (Fig. 2d, e). Furthermore, the apoptotic phenotype of MASTL depletion was rescued by the overexpression of mouse MASTL, excluding off-target effects of the siRNA (Fig. 2f). Thus, these results suggested that MASTL depletion induced cell death in breast cancer cell lines.

### MASTL depletion selectively reduces the oncogenic properties of breast cancer cells and does not affect the viability of normal cells

**Fig. 3:**
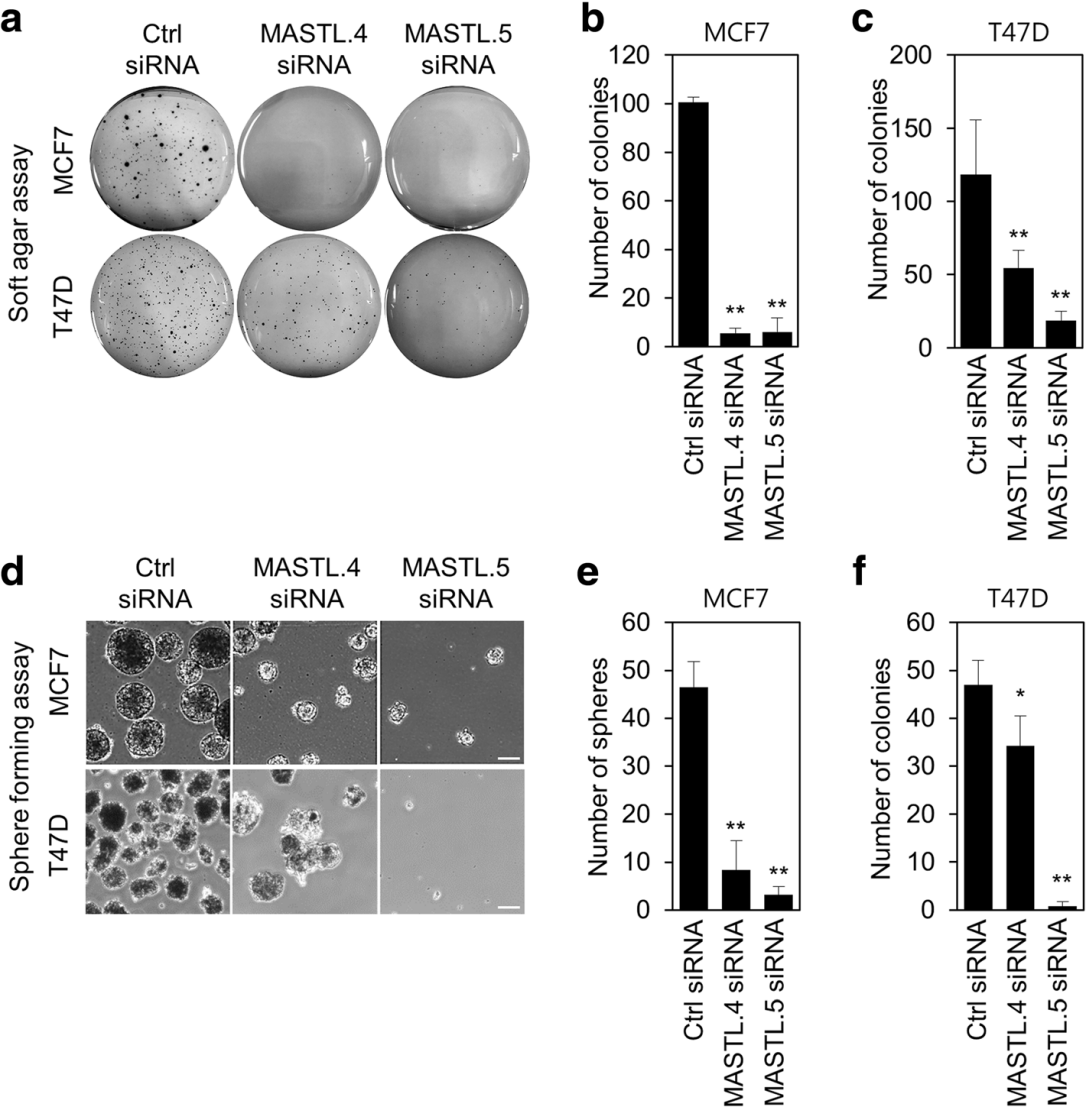
MASTL depletion reduces the oncogenic properties of breast cancer cells. MCF7 and T47D cells were transfected with either control siRNA, MASTL.4 siRNA, or MASTL.5 siRNA for 48 h. Colony formation was determined by using a soft agar assay by **a** representative images and **b**, **c** the quantification of colony formation of MCF7 and T47D cells. **d**, **e**, **f** Mammosphere formation was determined by using sphere formation assay by **d** representative images and **e**, **f** quantification of tumor sphere formation in MCF7 and T47D cells. Scale bars = 100 μm. The data represent typical results and are presented as the mean ± standard deviation of three independent experiments; **P < 0.01 and *P < 0.05

To examine the effect of MASTL depletion on the oncogenic properties of breast cancer cells, we first checked the effect of depletion on anchorage-independent cell growth. The soft agar assay showed that MASTL depletion by siRNA #4 and #5 decreased anchorage-independent cell growth in MCF7 and T47D cells (Fig. 3a-c). Furthermore, we evaluated the effect of depletion on the stemness of breast cancer cells. Interestingly, mammosphere formation was reduced after MASTL depletion by siRNA #4 and #5 siRNA in MCF7 and T47D cells (Fig. 3d-f), which suggested that MASTL inhibition was able to reduce the oncogenic properties of breast cancer cells with high MASTL expression.

**Fig. 4:**
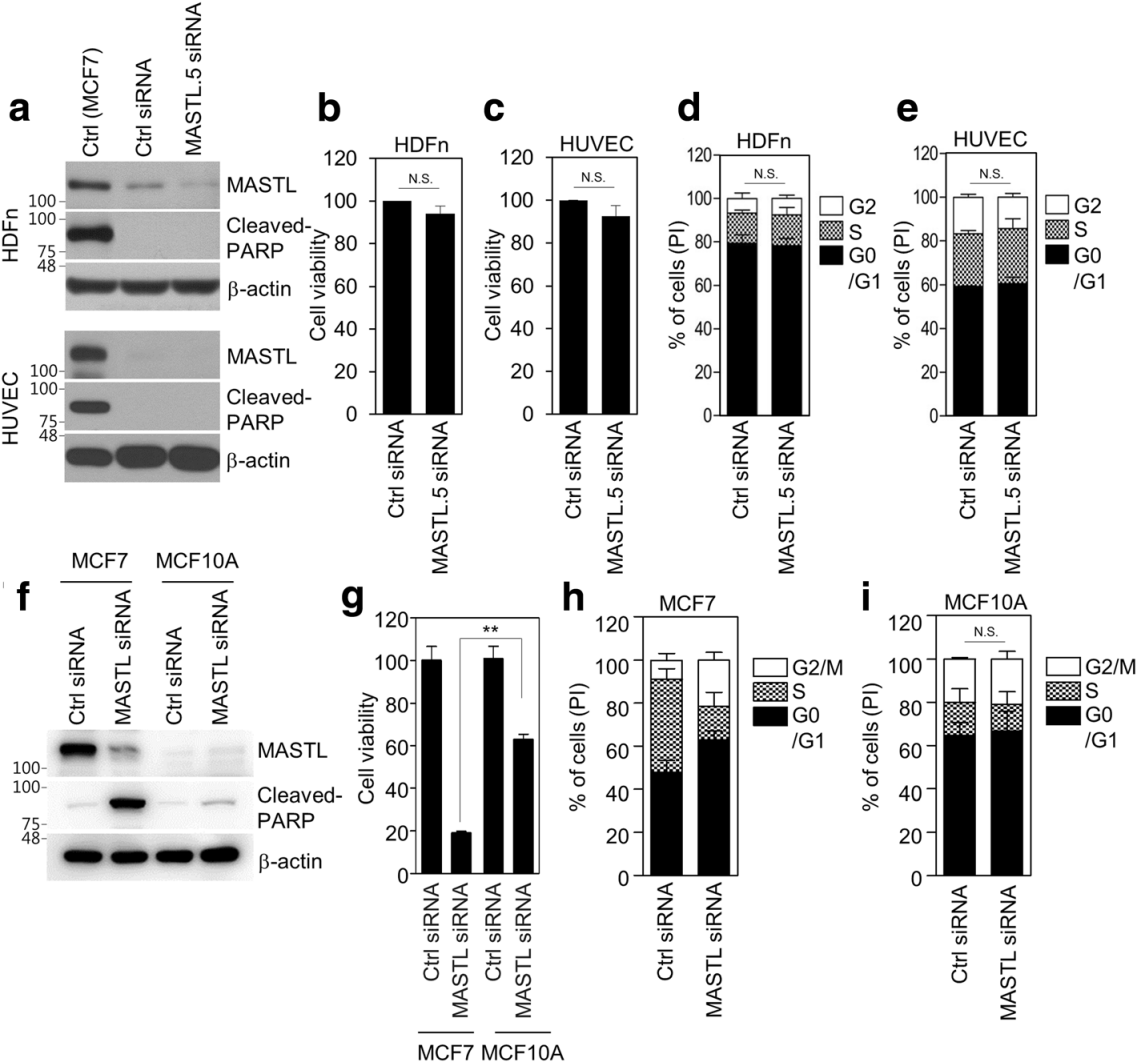
MASTL depletion does not affect the viability of normal cells. HUVEC, HDFn, MCF7, and MCF10A cells were transfected with either control siRNA or MASTL.5 siRNA for 48 h. **a**, **f** The cells were analyzed by immunoblotting with the indicated antibodies. **b**, **c**, **g** Cell viability was determined by WST-8 assay. **d**, **e**, **h**, **i** The cell cycle was analyzed by FACS. The data represent typical results and are presented as the mean ± standard deviation of three independent experiments; **P < 0.01. N.S.: not significant

As the inhibition of PLK1 does not induce apoptosis in non-transformed normal cells [3], we then examined whether MASTL depletion induced cell death in non-transformed normal cells and normal breast cells. Two types of human non-transformed normal cells, human dermal fibroblasts, HDFn, and human umbilical vein endothelial cells, HUVECs, of different origins were used. First, we determined MASTL expression in two non-transformed normal cell lines. Irradiated MCF7 cells, which overexpress MASTL and cleaved-PARP, were used as a positive control. MASTL expression in the two normal cell lines was very low compared to that in the positive control MCF7 cells (Fig. 4a). Similar to the effects of PLK1 inhibition in normal cells [3], MASTL depletion did not induce cell death (Fig. 4a) or affect the viability (Fig. 4b, c) and cell cycle (Fig. 4d, e) of HDFn and HUVEC cells. In addition, we also observed that MASTL depletion was much less sensitive to normal breast MCF10A cells compared to that of MCF7 cells, as determined by the levels of cleaved-PARP (Fig. 4f), cell viability assay (Fig. 4g), and cell cycle analysis (Fig. 4h, i). Therefore, our results suggested that MASTL inhibition selectively reduced the oncogenic properties of breast cancer cells and did not affect the viability of normal cells.

### MASTL depletion causes mitotic cell death through PP2A activation

**Fig. 5:**
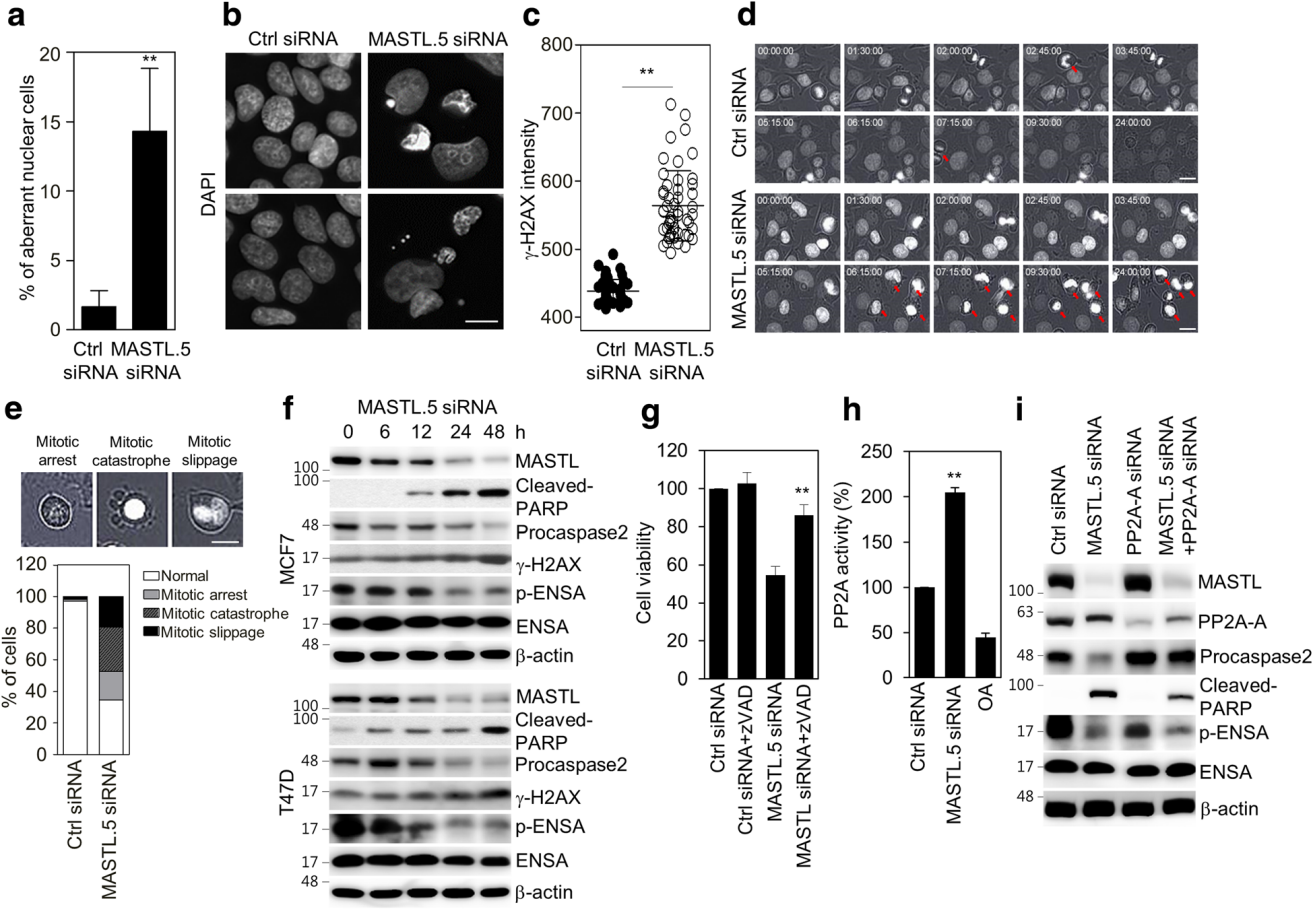
MASTL depletion causes mitotic catastrophe. MCF7 cells were transfected with either control siRNA or MASTL.5 siRNA for 48 h. **a** MASTL-depleted cells were scored for abnormal mitosis (≥ 200 mitotic cells for each data point, *n* = 4, error bars show ± SD). **b** Representative images of MASTL-depleted cells stained with DAPI (blue). **c** The intensities of γ-H2AX were scanned and determined by IN Cell Analyzer HCA System. The intensities of 50 dots in the control and MASTL-depleted cells were determined by the γ-H2AX intensity from 200 ~ 700 cells. **d** The time of mitotic entry was determined by observing mitotic cell rounding with signs of DNA condensation (red arrow) and was monitored by time-lapse microscopy. **e** After control (Ctrl) siRNA or MASTL.5 siRNA transfection for 24 h, the cells were quantified in four statuses (normal, mitotic arrest, mitotic catastrophe, and mitotic slippage). **f** MCF7 and T47D cells were transfected with either control siRNA or MASTL.5 siRNA for the indicated time periods. The cells were analyzed by immunoblotting with the indicated antibodies. **g** MCF7 cells were transfected with either 5 nmol/l control siRNA or 5 nmol/l MASTL.5 siRNA and then incubated without or with zVAD for 48 h. The cell viability was determined by WST-8 assay. **h** PP2A-Aα/β proteins were immunoprecipitated using an anti-PP2A-Aα/β antibody and analyzed for PP2A activity. **i** MCF7 cells were transfected with the indicated siRNAs targeting MASTL.5 or PP2A-Aα/β for 48 h. The cells were analyzed by immunoblotting with the indicated antibodies. The data represent typical results and are presented as the mean ± standard deviation of three independent experiments; Scale bars = 10 μm. **P < 0.01

As MASTL depletion induces G_2_ arrest and multiple mitotic defects in somatic cells [12], we assumed that MASTL depletion induced mitotic cell death in breast cancer cells. To test this assumption, we first examined mitotic defects in MASTL-depleted MCF7 cells. We also observed that MASTL depletion increased mitotic arrest and the accumulation of phospho-histone H3 (Ser10) in MCF7 cells (Additional file 2: Figure S2a). In addition, MASTL depletion increased aberrant nuclei, such as micro-nuclei and multi-nuclei (Fig. 5a, b), mitotic defects (Additional file 2: Figure S2b), and phospho-histone H2AX (Ser139) (γ-H2AX), a marker of DNA damage [24] (Fig. 5c), which are common phenotypic characteristics of mitotic catastrophe [6]. Furthermore, time-lapse microscopic analysis indicated that MASTL depletion dramatically increased the number of cells in abnormal mitotic arrest (Fig. 5d). Of these arrested cells, 28% showed highly condensed chromatin and membrane blebbing, which indicated mitotic catastrophe; the 18% were still arrested, but 19% bypassed cytokinesis (mitotic slippage), resulting in polyploidy (Fig. 5e). In support of these results, western blotting showed that MASTL depletion increased cleaved-PARP and γ-H2AX, and activated caspase-2, as evidenced by the decrease in procaspase-2, which is a major caspase in the mitotic cell death in response to DNA damage [7], and decreased phosphorylated ENSA, a substrate of MASTL, in a time-dependent manner in MCF7 and T47D cells (Fig. 5f). In addition, we confirmed that MASTL-induced mitotic cell death was rescued by treatment with zVAD, a pan-caspase inhibitor (Fig. 5g), which supported the dependency of MASTL depletion-induced mitotic cell death on caspase activation. Next, we determined PP2A activity in MASTL-depleted cells, since MASTL inhibits PP2A activity during mitosis in various model systems [12, 16, 25, 26], and found that MASTL depletion increased PP2A activity (Fig. 5h). Moreover, we found that MASTL-depletion-induced mitotic cell death was partially blocked by PP2A inhibition from PP2A-Aα/β siRNA (Fig. 5i). Therefore, our data suggested that MASTL inhibition was able to induce mitotic cell death through PP2A activation in breast cancer cells.

### MASTL depletion enhances radiosensitivity of breast cancer cells and sensitizes radioresistant breast cancer stem cells

**Fig. 6:**
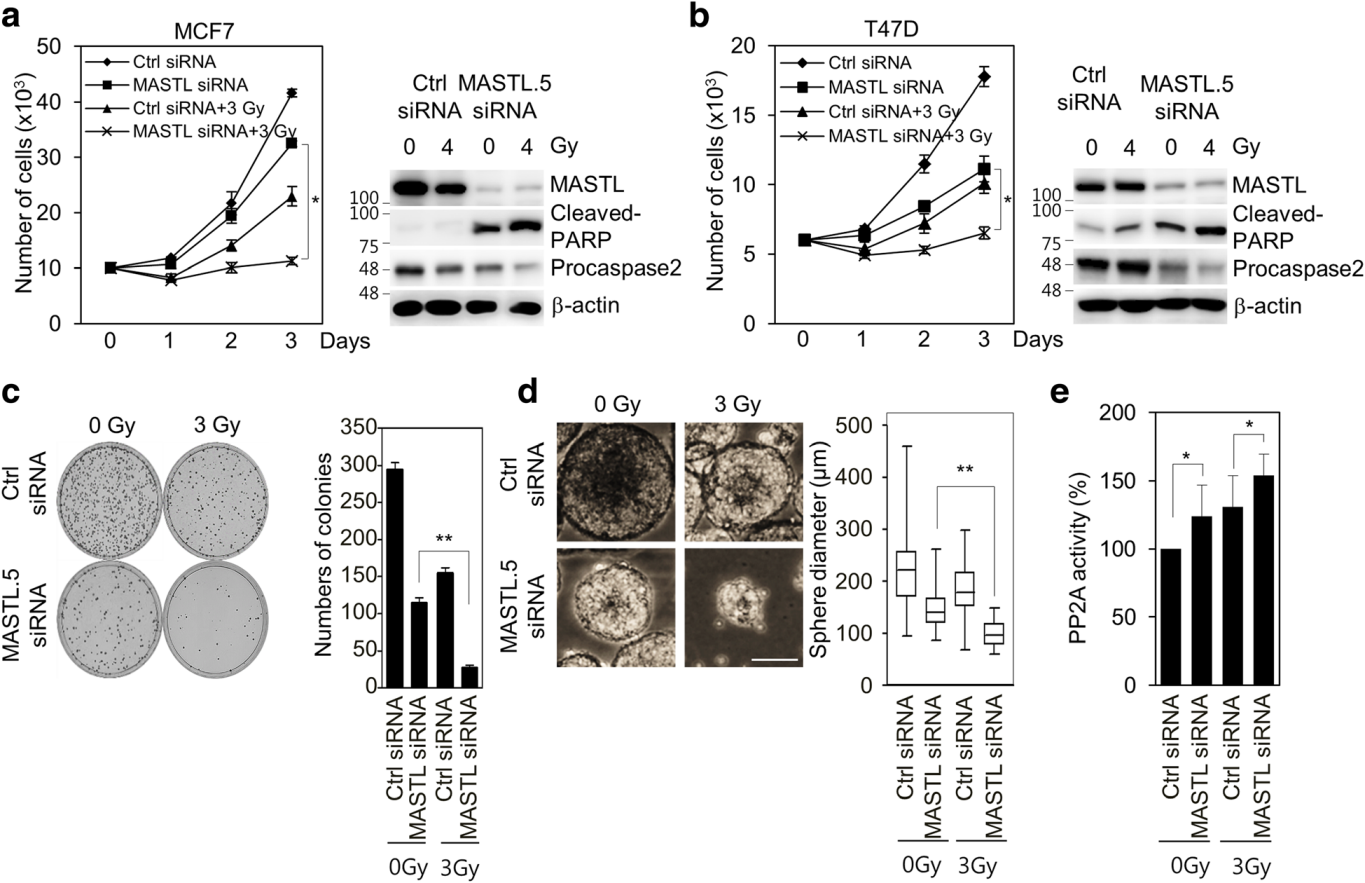
MASTL depletion increases the radiosensitivity of breast cancer cells. MCF7 cells were transfected with either 5 nmol/l control siRNA or MASTL.5 siRNA. Cells were treated 0, 3 or 4 Gy irradiation for 42 h. **a**, **b** Cell proliferation was determined by FACS analysis and then analyzed by immunoblotted with the indicated antibodies. **c** The results of the clonogenic assay. Representative images of the cells treated the indicated conditions (left panel). The number of colonies was measured (right panel). **d** The sphere forming assay was performed (> 110 sphere colonies per data point). Scale bar = 100 μm. Representative images of sphere forming assay. **e** PP2A-Aα/β proteins were immunoprecipitated using an anti-PP2A-Aα/β antibody and analyzed for PP2A activity. The data represent typical results and are presented as the mean ± standard deviation of three independent experiments; **P < 0.01 and *P < 0.05

As mitotic cell death is the major form of radiation-induced cell death and the induction of mitotic cell death is important for enhancing the efficacy of radiotherapy [6, 8], we further examined the effects of MASTL depletion on radiosensitivity in breast cancer cells and radioresistant breast cancer stem cells. In the experiments, 5 nmol/l control siRNA or MASTL siRNA #5 was used to examine the combined effect of MASTL inhibition and irradiation. In MCF7 and T47D cells, we observed that MASTL depletion efficiently reduced cell proliferation, increased cleaved-PARP, and decreased procaspase-2 in response to irradiation (Fig. 6a, b). In addition, clonogenic analysis also indicated that MASTL depletion decreased colony formation of MCF7 and T47D cells (Fig. 6c and Additional file 3: Figure S3a), which indicated that MASTL inhibition enhanced the radiosensitivity of breast cancer cells. As cancer stem cells are a key factor in the radioresistance of breast cancer cells [9], we also determined if MASTL inhibition modulated the stemness of breast cancer cells in response to irradiation. We found that mammosphere formation was reduced in the MASTL-depleted MCF7 and T47D cells in response to irradiation (Fig. 6d and Additional file 3: Figure S3b), which suggested that MASTL inhibition was able to reduce breast cancer stemness in response to irradiation. Moreover, we also observed that MASTL depletion increased the radiation-induced PP2A activation (Fig. 6e), suggesting that MASTL inhibition enhances radiosensitivity of breast cancer cells through PP2A activation.

**Fig. 7:**
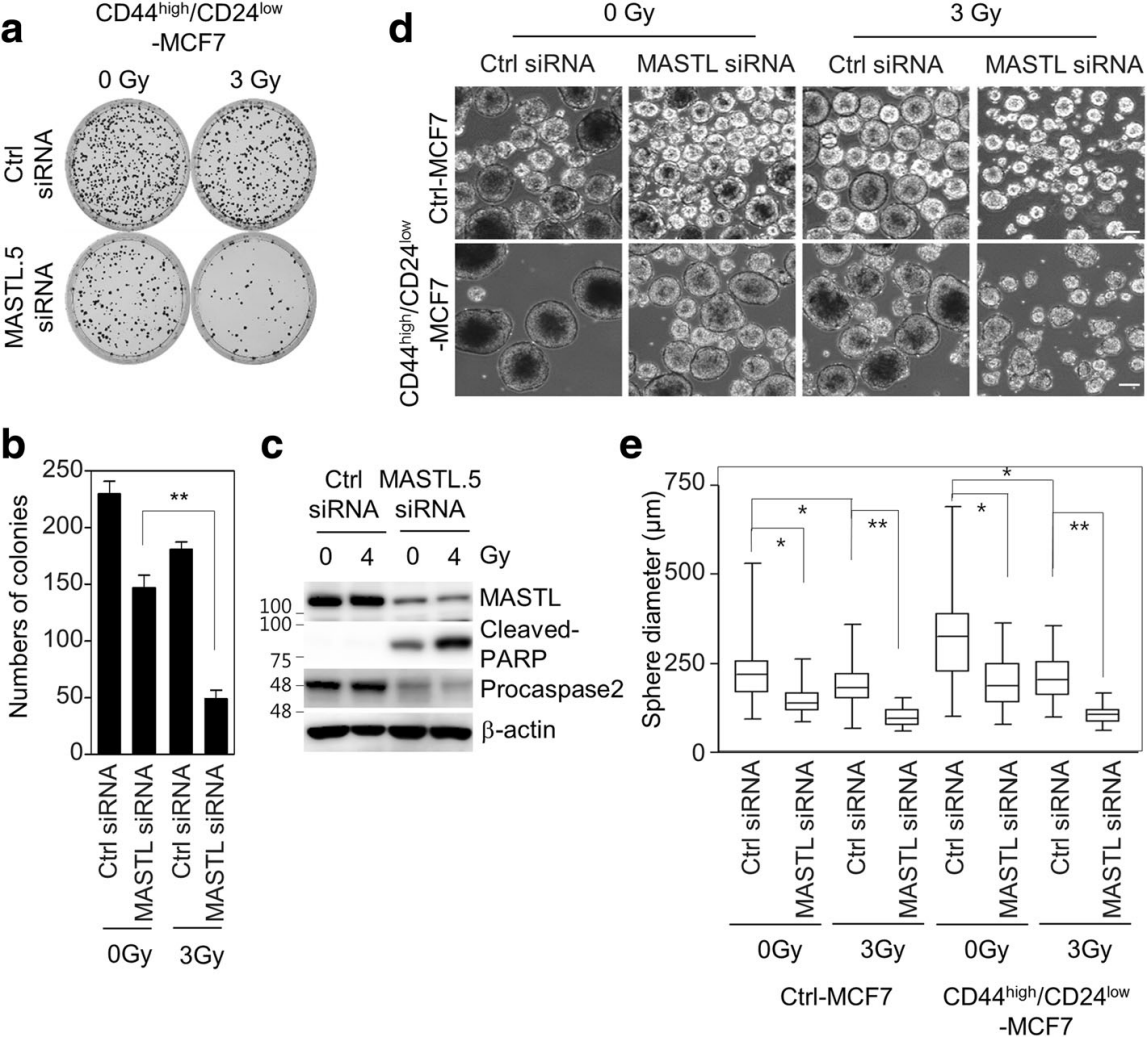
MASTL depletion sensitizes radioresistant breast cancer stem cells. CD44^high^/CD24^low^ MCF7 cells were transfected with either 5 nmol/l control siRNA or MASTL.5 siRNA. The cells were irradiated with 0, 3, or 4 Gy for 42 h. **a**, **b** The clonogenic assay results. **a** Representative images of the cells treated with the indicated conditions. **b** The number of colonies was measured. **c** The cells were analyzed by immunoblotting with the indicated antibodies. **d**, **e** The sphere forming assay was performed. Scale bars = 100 μm. **d** Representative images of sphere forming assay in Ctrl MCF7 and CD44^high^/CD24^low^ MCF7 cells. **e** The sphere forming capacity was measured from the sphere diameter (μm) (> 110 sphere colonies per data point). The data represent typical results and are presented as the mean ± standard deviation of three independent experiments; **P < 0.01 and *P < 0.05

To further examine the role of MASTL in the radioresistance associated with breast cancer stem cells (BCSCs), we sorted BCSCs from MCF7 cells by using CD44^high^/CD24^low^, a marker for BCSCs [27]. We have previously demonstrated that CD44^high^/CD24^low^ MCF7 cells are breast cancer stem cells with a radioresistant phenotype [19]. MASTL depletion significantly reduced the colony formation of CD44^high^/CD24^low^ MCF7 cells in response to irradiation (Fig. 7a, b). Similarly, MASTL depletion increased cleaved-PARP and decreased procaspase-2 in CD44^high^/CD24^low^ MCF7 cells (Fig. 7c). Furthermore, MASTL depletion significantly reduced the mammosphere formation of CD44^high^/CD24^low^ MCF7 cells in response to irradiation, in which the inhibition rate was more significant than the inhibition rate of the MASTL-depleted control MCF7 cells (Fig. 7d, e). Collectively, our data suggested that MASTL inhibition was able to suppress the radioresistance of breast cancer stem cells through the promotion of radiation-induced mitotic catastrophe.

## Discussion

Although several studies have recently reported that MASTL inhibition reduces tumor growth in vitro and in vivo [13–16], the antitumor mechanism of MASTL targeting in breast cancer cells is still unclear. Our data showed that MASTL targeting reduced breast cancer cell growth through the promotion of mitotic catastrophe by PP2A activation. However, MASTL inhibition did not induce mitotic catastrophe in non-transformed normal cells with low MASTL expression. In addition, MASTL inhibition enhanced the radiosensitivityof breast cancer cells through a reduction in the formation of cancer stemness. Therefore, our data provided evidence that MASTL targeting is not only an attractive target for selective antitumor treatment, but also for a combination treatment with radiotherapy.

Recent studies showed that MASTL is overexpressed in various cancer tissues, including breast, head and neck, and colorectal cancers [13, 14, 16]. We also observed consistently increased MASTL expression in breast cancer tissues and cell lines that was correlated with tumor stage and poor prognosis. Similarly, the mitotic kinase PLK1 is also overexpressed in various cancer tissues, including breast cancer, and frequently associated with poor prognosis [4, 28]. Given that cancer cells are actively proliferating with increased numbers of mitotic cells, it appears that increased MASTL in various cancers is associated with the increased mitotic activity of cancer cells. However, recent reports showed that MASTL was able to promote cell transformation through the modulation of Akt activity, whereas most of the mitotic kinases, including Plk1, Aurora A/B, and Nek2, are unable to induce cellular transformation in human malignancies [14]. Indeed, our observation that MASTL inhibition reduced oncogenic properties, as evidenced by the soft agar assay and the sphere formation assay, also supported the capability of MASTL to promote cell transformation. As the evasion of mitotic catastrophe is a step in tumor development [6], it is possible that the increases in MASTL found in various types of cancers are associated not only with mitotic activity, but also with the oncogenic capacity of MASTL, which could be linked to the suppression of mitotic catastrophe. However, the exact mechanisms of MASTL-mediated cellular transformation are unclear and should be defined in future studies.

Although several recent studies indicated that MASTL inhibition reduced cancer cell proliferation, the selective nature of the antitumor mechanism of MASTL inhibition in cancer cells is poorly understood. Our data showed that MASTL depletion preferentially induced cancer cell death in breast cancer cells with high MASTL expression, whereas MASTL depletion did not affect the viability of normal cell lines with almost non-existent expression of MASTL (Fig. 4), which implied that breast cancer cells with high MASTL expression may be reliant on MASTL overexpression for survival and proliferation. As oncogene addiction is important factor for the development of molecularly targeted drugs [29], it is possible that the selection of tumor patients based on the MASTL biomarker could be useful for decisions on MASTL treatment.

Mitotic catastrophe acts as an oncosuppressive mechanism to sense mitotic failure and directs the cell to apoptosis, necrosis, or senescence to prevent genomic instability [6]. Thus, the evasion of mitotic catastrophe could be associated with a gateway for malignant transformation and tumor development [6]. Notably, several inducers of mitotic catastrophe, such as radiation, are less effective in non-transformed fibroblasts than in cancer cells [1, 6], which suggests that induction of mitotic catastrophe could be an advantage for the selective eradication of cancer cells. Thus, it appears that the different sensitivity of MASTL inhibition between breast cancer cells and normal cells is associated with the selective induction of mitotic catastrophe between cancer cells and normal cells, which have a greater tolerance. In addition, we determined that MASTL depletion-induced mitotic cell death was dependent on PP2A activation. Similarly, other reports have shown that MASTL-null mouse embryonic fibroblasts cells underwent mitotic collapse through defective chromosome condensation and prometaphase arrest; this phenotype was rescued by the co-depletion of PP2A-B55 regulatory subunits [30]. Furthermore, a recent report also suggested that the depletion of B55α and B55δ, a subunit of PP2A, partially rescued the defective phenotype of MASTL-depleted cells [16]. Thus, this implied that MASTL inhibition induced mitotic cell death not only through the activation of PP2A, but also by other factors. As PP2A is responsible for the dephosphorylation of many oncogenic substrates, including Akt, mTOR1, and ERK1/2, it may be possible that the partial rescue of the MASTL inhibition phenotype by PP2A may be related to the abundance of PP2A substrates.

As irradiation induces G_2_/M mitotic arrest and promotes mitotic cell death in cancer cells, radiation-induced mitotic cell death is important to enhance the efficacy of radiotherapy [8]. MASTL was reported to regulate the DNA damage response in *Xenopus* and head and neck cancer cells [13, 18]. In addition, a genome-wide siRNA screen identified MASTL as a potential target for radiosensitization in non-small cell lung cancer cells [31]. Moreover, cancer stemness is closely associated with radioresistance and enhanced DNA repair capacity in several cancer cells, including breast cancer [9]. Notably, our data showed that MASTL inhibition-mediated mitotic cell death enhanced the radiosensitivity of breast cancer cells through a reduction in the formation of cancer stem cells and that MASTL inhibition increased DNA damage. Therefore, it is likely that MASTL inhibition may be a potential strategy for selective anticancer treatment and a potential therapeutic combination target with radiotherapy through the promotion of mitotic catastrophe.

## Conclusions

Our data showed that MASTL inhibition induced mitotic catastrophe through PP2A activation; in turn, this preferentially inhibited cancer growth and enhanced the radiosensitivity of breast cancer cells. Our study provides support for the use of MASTL-specific inhibitors as tumor-selective drugs and in combination with radiotherapy through the promotion of mitotic catastrophe.

## Additional files

Additional file 1: **Figure S1.** MASTL is associated with poor prognosis in breast cancer. The survival of MASTL in breast cancer was analyzed by using the PROGgene database. **a** Kaplan-Meyer analysis of overall survival in GSE37751 and GSE42568 datasets, **b** recurrence-free survival in GSE4922 and GSE6532 datasets, and **c** metastasis-free survival in GSE48408 and GSE6532 datasets. Survival analysis was performed using a log-rank test. *P < 0.05. (TIF 623 kb)

Additional file 2: **Figure S2.** MASTL depletion increases G2 arrest and the accumulation of pH 3. **a** The quantification of the relative percentage of cells expressing red fluorescence (pH 3). **b** Representative images of a normal mitotic cells (left panel) and MASTL-depleted mitotic defect cells stained with anti-acetyl-tubulin antibody (green), anti-phospho-Histone H3 antibody (red), and DAPI (blue). Scale bar = 10 μm. (TIF 763 kb)

Additional file 3: **Figure S3.** MASTL depletion increases the radiosensitivity of T47D breast cancer cells. T47D cells were transfected with either 5 nmol/l control siRNA or MASTL.5 siRNA. The cells were irradiated with 0, 3, or 4 Gy irradiation for 42 h. **a** The clonogenic assay results. Representative images of the cells treated the indicated conditions (left panel). The number of colonies was measured (right panel). **b** The sphere forming assay was performed. Scale bar = 100 μm. Representative images of sphere forming assay (left panel). The sphere forming capacity was measured from the sphere diameter (μm) (right panel). The data represent typical results and are presented as the mean ± standard deviation of three independent experiments; **P < 0.01 and *P < 0.05. (TIF 830 kb)

## Abbreviations

BCSCs: Breast cancer stem cells
ENSA: α-endosulfine
MASTL: Microtubule-associated serine/threonine kinase-like
PLK1: Polo-like kinase 1
PP2A: Protein phosphatase 2A
siRNA: Small interfering RNA
UTR: Untranslated region
