# Supplementary figures and images for: MASTL inhibition promotes mitotic catastrophe through PP2A activation to inhibit cancer growth and radioresistance in breast cancer cells
Source: BMC Cancer. 2018 Jul 5;18:716. doi: 10.1186/s12885-018-4600-6 (PMC6034325; doi:10.1186/s12885-018-4600-6)

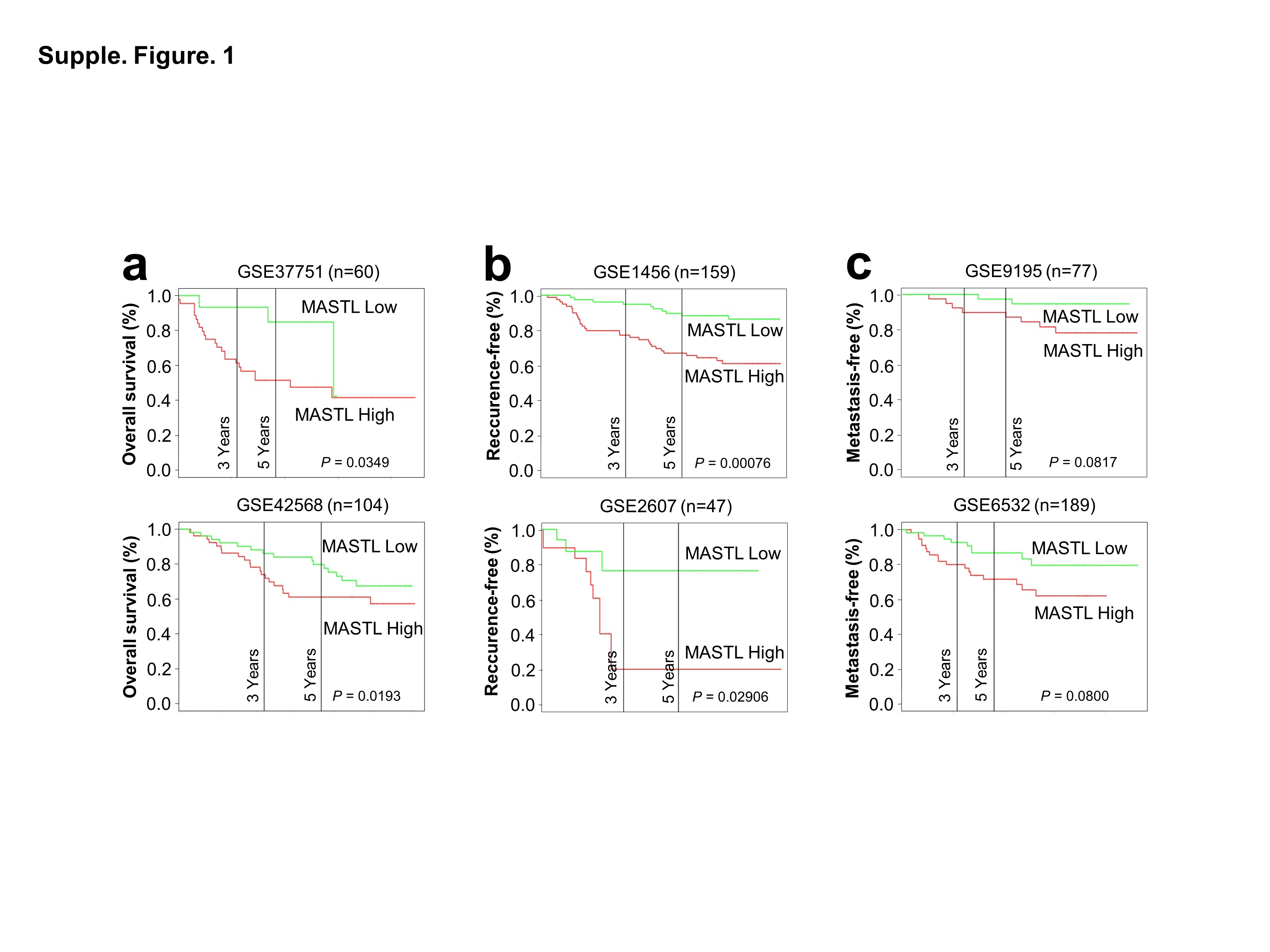

Supplement: Supplementary file 1 — Figure S1. MASTL is associated with poor prognosis in breast cancer. The survival of MASTL in breast cancer was analyzed by using the PROGgene database. a Kaplan-Meyer analysis of overall survival in GSE37751 and GSE42568 datasets, b recurrence-free survival in GSE4922 and GSE6532 datasets, and c metastasis-free survival in GSE48408 and GSE6532 datasets. Survival analysis was performed using a log-rank test. *P < 0.05. (TIF 623 kb) [file 12885_2018_4600_MOESM1_ESM.tif]

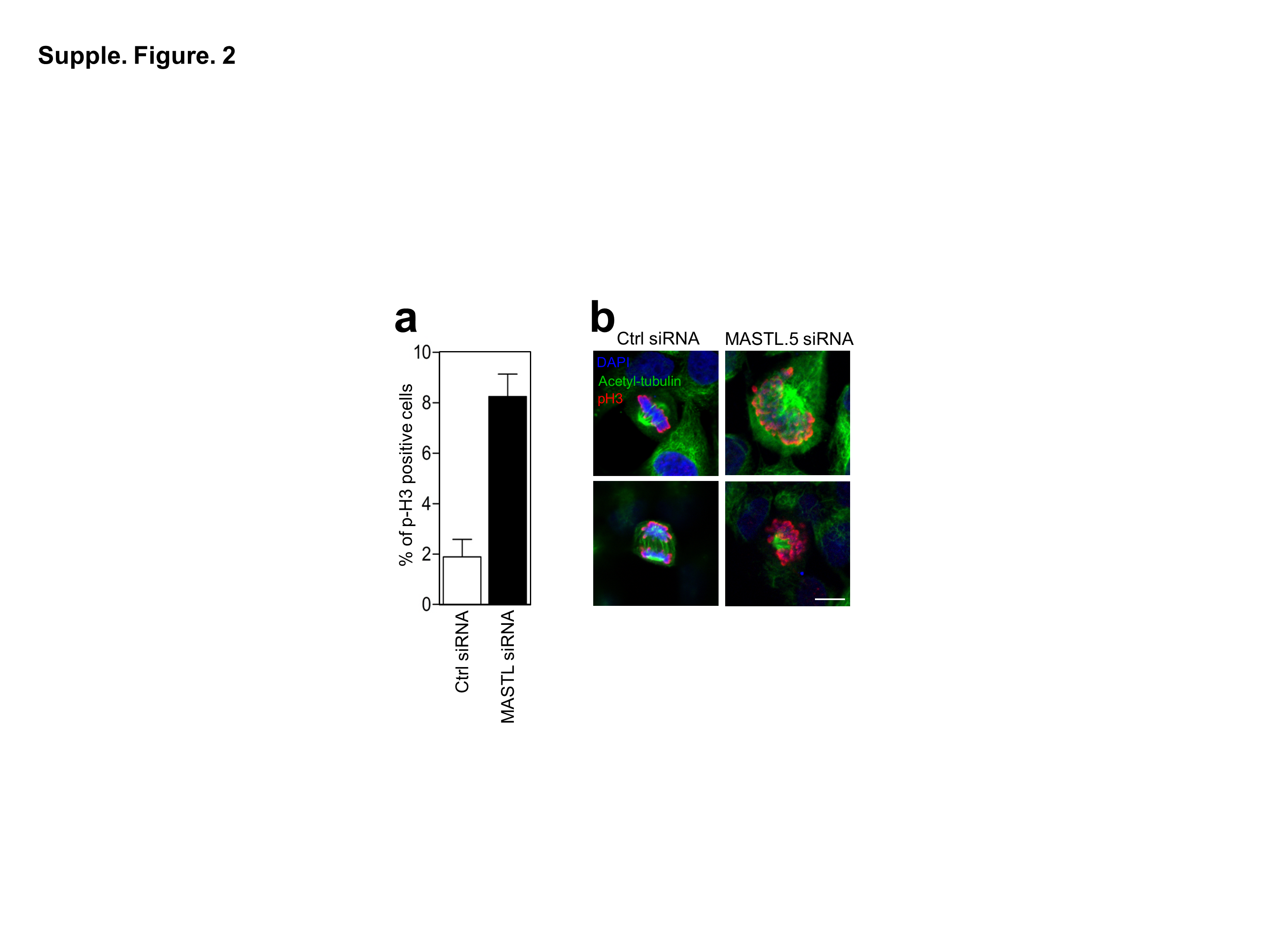

Supplement: Supplementary file 2 — Figure S2. MASTL depletion increases G2 arrest and the accumulation of pH 3. a The quantification of the relative percentage of cells expressing red fluorescence (pH 3). b Representative images of a normal mitotic cells (left panel) and MASTL-depleted mitotic defect cells stained with anti-acetyl-tubulin antibody (green), anti-phospho-Histone H3 antibody (red), and DAPI (blue). Scale bar = 10 μm. (TIF 763 kb) [file 12885_2018_4600_MOESM2_ESM.tif]

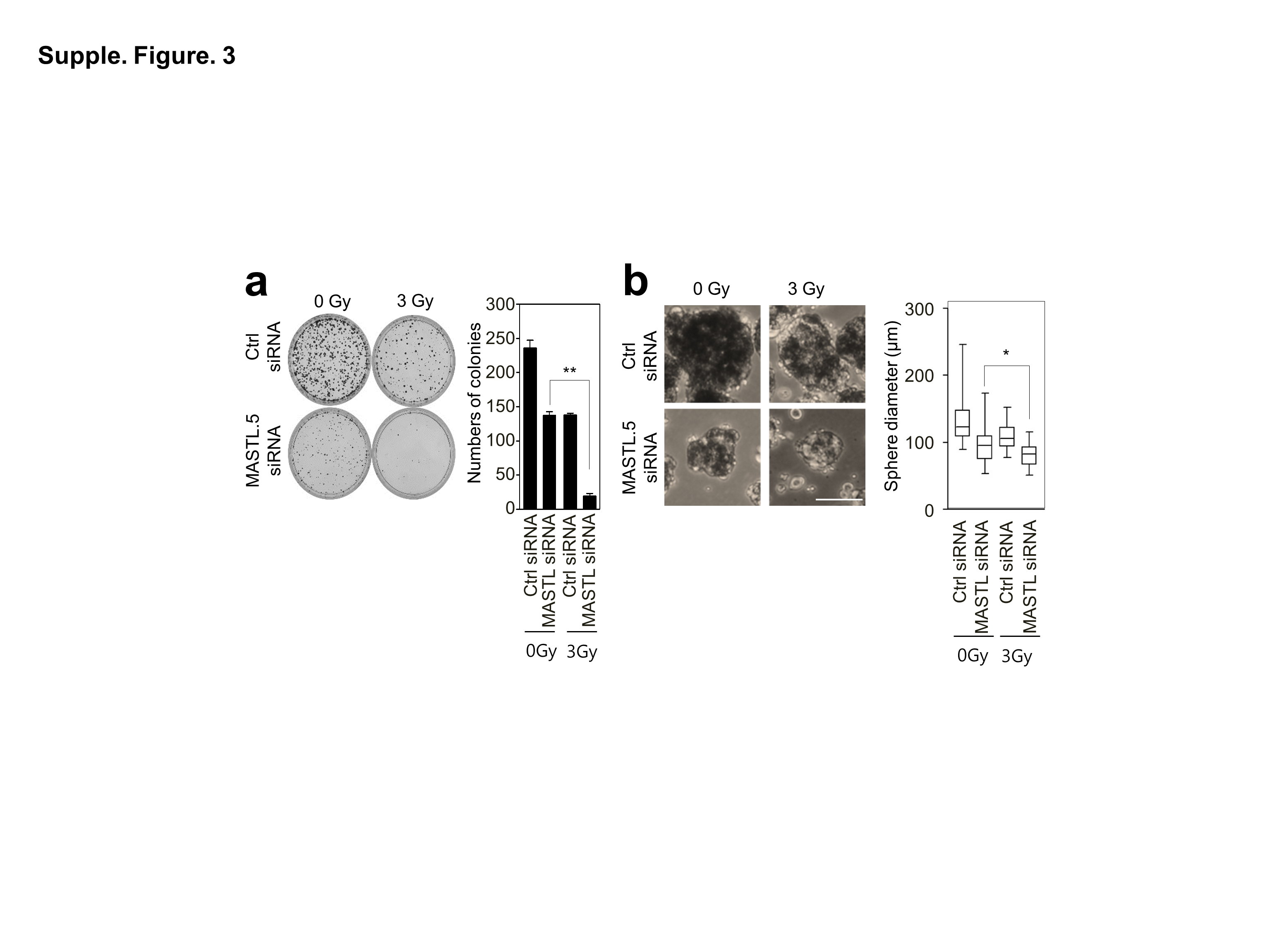

Supplement: Supplementary file 3 — Figure S3. MASTL depletion increases the radiosensitivity of T47D breast cancer cells. T47D cells were transfected with either 5 nmol/l control siRNA or MASTL.5 siRNA. The cells were irradiated with 0, 3, or 4 Gy irradiation for 42 h. a The clonogenic assay results. Representative images of the cells treated the indicated conditions (left panel). The number of colonies was measured (right panel). b The sphere forming assay was performed. Scale bar = 100 μm. Representative images of sphere forming assay (left panel). The sphere forming capacity was measured from the sphere diameter (μm) (right panel). The data represent typical results and are presented as the mean ± standard deviation of three independent experiments; **P < 0.01 and *P < 0.05. (TIF 830 kb) [file 12885_2018_4600_MOESM3_ESM.tif]
